# FOXD1‐AS1 regulates FOXD1 translation and promotes gastric cancer progression and chemoresistance by activating the PI3K/AKT/mTOR pathway

**DOI:** 10.1002/1878-0261.12728

**Published:** 2020-11-14

**Authors:** Qiong Wu, Jiali Ma, Jue Wei, Wenying Meng, Yugang Wang, Min Shi

**Affiliations:** ^1^ Department of Gastroenterology Tongren Hospital Shanghai Jiao Tong University School of Medicine China

**Keywords:** eIF4G‐eIF4E‐eIF4A translational complex, FOXD1, FOXD1‐AS1, gastric cancer, PI3K/AKT/mTOR pathway

## Abstract

Gastric cancer (GC) is a common gastrointestinal cancer with a high global mortality. Recent reports have suggested that long noncoding RNA (lncRNA) are implicated in multiple aspects of GC, including pathogenesis, progression, and therapeutic response. Herein, we investigated the function of FOXD1‐AS1 in GC progression and chemoresistance. Expression of FOXD1‐AS1 was low in normal stomach tissues but was upregulated in GC cell lines. Silencing of FOXD1‐AS1 impaired GC cell proliferation and motility *in vitro*, and repressed tumor growth and metastasis *in vivo*. Importantly, FOXD1‐AS1 upregulation increased the resistance of GC cells to cisplatin. Moreover, we found that FOXD1‐AS1 promoted FOXD1 protein translation through the eIF4G‐eIF4E‐eIF4A translational complex. We also demonstrated that FOXD1‐AS1 released eIF4E from phosphorylated 4E‐BP1 and thereby strengthened the interaction of eIF4E with eIF4G by activating the PI3K/AKT/mTOR pathway. Activation of the PI3K/AKT/mTOR pathway was due to the post‐transcriptional upregulation of PIK3CA, in turn induced by FOXD1‐AS1‐mediated sequestering of microRNA (miR)‐466. Furthermore, we verified that FOXD1‐AS1 facilitated GC progression and cisplatin resistance in a FOXD1‐dependent manner. In conclusion, FOXD1‐AS1 aggravates GC progression and chemoresistance by promoting FOXD1 translation via PIK3CA/PI3K/AKT/mTOR signaling. These findings highlight a novel target for treatment of patients GC, particularly patients with cisplatin resistance.

## Graphical abstract

Cytoplasmic FOXD1‐AS1 sponges miR‐466 to increase PIK3CA expression, so as to activate the PI3K/AKT/mTOR signaling pathway and induce 4E‐BP1 phosphorylation, therefore leading to enhanced interaction of eIF4E with eIF4G, and resulting in boosted protein levels of FOXD1, which finally facilitate GC cell growth, metastasis, and chemoresistance.

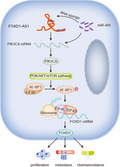

**Abbreviations**
ANOVA: analysis of variance
CCK‐8: cell counting kit 8
ce, competing endogenous
Co‐IP: co‐immunoprecipitation
DAPI: 4′,6‐diamidino‐2‐phenylindole
DDP: cis‐dichlorodiamine platinum(II)
EdU: 5‐ethynyl‐2′‐deoxyuridine
FOXD1: Forkhead box D1
FOXD1‐AS1: Forkhead box D1 antisense RNA 1
GAPDH: glyceraldehyde‐3‐phosphate dehydrogenase
GC: gastric cancer
IHC: immunohistochemical
ISH: in  situ hybridization
LncRNA: long noncoding RNA
miR, microRNA
qRT–PCR: quantitative real‐time polymerase chain reaction
RIP: RNA immunoprecipitation
sh: short hairpin
TdT: , terminal deoxynucleotidyl transferase
TUNEL: TdT; dUTP nick end labeling
U6: small nuclear RNA U6

## 1 Introduction

Gastric cancer (GC) is one of the most frequent types of gastrointestinal cancer and is the leading cause of cancer‐associated death worldwide (McLean and El‐Omar, 2014; Torre *et al*., 2015). Nowadays, surgery is still the only curative strategy for patients with gastric carcinoma *in situ*, but most GC patients are not suitable for surgical resection since they are definitely diagnosed at advanced stage (Dassen *et al*., 2013; Karimi *et al*., 2014). For these patients, chemotherapy becomes the first‐line therapeutic strategy to inhibit tumor development. Unfortunately, despite the advances in developing chemotherapeutic drugs, the efficacy of chemotherapy is still disappointing in advanced GC patients with the acquired chemoresistance (Cunningham *et al*., 2006; Sakuramoto *et al*., 2007). To improve clinical outcomes of GC patients, identification of the mechanisms underlying carcinogenesis and chemoresistance is quite urgent.

Long noncoding RNA (lncRNA) are transcripts belonging to noncoding RNA over 200 nucleotides in length (Esteller, 2011). In recent decades, increasing studies have indicated the importance of lncRNA in the regulation of cancer initiation and progression (Huarte, 2015; Prensner and Chinnaiyan, 2011; Schmitt and Chang, 2016). Additionally, many lncRNA have been reported to play crucial roles in the tumorigenesis and development of GC, e.g. GClnc1 (Sun *et al*., 2016b), LINC01234 (Chen *et al*., 2018), PVT1 (Xu *et al*., 2017), HOXA11‐AS (Sun *et al*., 2016a), GAPLINC (Hu *et al*., 2014), and BC032469 (Lü *et al*., 2015). Accordingly, lncRNA have also been revealed to accelerate chemoresistance of GC cells over decades (Jiang *et al*., 2019; YiRen *et al*., 2017). FOXD1 antisense RNA 1 (FOXD1‐AS1) is a newly recognized lncRNA that has been recently proved to be an oncogenic biomarker in glioma (Gao *et al*., 2020). However, the probable role of FOXD1‐AS1 in other cancers, including GC, remains to be substantiated.

In current work, we planned to investigate the functional role and potential molecular mechanism of FOXD1‐AS1 in the tumorigenesis and chemoresistance of GC.

## 2 Materials and methods

### 2.1 Bioinformatics analysis

The expression of FOXD1‐AS1 in 53 types of human normal tissues was obtained from UCSC (http://genome.ucsc.edu/). The possible binding sites of micro (mi)R‐466 in FOXD1‐AS1 and PIK3CA sequences were obtained from lncBase v.2 from DIANA tool (http://carolina.imis.athena‐innovation.gr/diana_tools/web/index.php?r=lncbasev2%2Findex) and targetscan (http://www.targetscan.org/vert_72/), respectively.

### 2.2 Cell culture

Human normal gastric epithelial cells (GES1), GC cells (BGC‐823, MKN28, MGC803, MKN45, AGS), and human embryonic kidney cells (HEK‐293T) were obtained from the Shanghai Cell Bank of the Chinese Academy of Sciences (China). GES‐1 and GC cells were cultured in RPMI‐1640 medium (Thermo Fisher Scientific, Waltham, MA, USA) containing 10% heat‐inactivated FBS (Thermo Fisher Scientific) and 1% penicillin and streptomycin (Thermo Fisher Scientific). HEK‐293T cells were grown in DMEM (Thermo Fisher Scientific) containing 1% penicillin/streptomycin plus 10% FBS. Cells were cultured under standard conditions (5% CO_2_ and 37 °C) with a replacement of medium every 3 days.

### 2.3 Cell treatment

Treatment with cisplatin (DDP; Sigma‐Aldrich, St. Louis, MO, USA) was performed to construct the DDP‐resistant cells (BGC‐823R and MKN28R). In detail, the parental BGC‐823 and MKN28 cells underwent DDP treatment with slowly increasing concentrations of DDP. When cells could tolerate the treatment of DDP with an IC_50_concentration for 6 months, the resistant cells (BGC‐823R and MKN28R) were successfully established. Thereafter, BGC‐823R and MKN28R cells were maintained in 1 μg·mL^−1^ of DDP for subsequent use, and the sensitivity of these cells to DDP was routinely examined every 2 months. In addition, the resistant cells were grown in DDP‐free medium for 3 days before use. BGC‐823 and MKN28 cells under transient DDP treatment (with 1 μg·mL^−1^ of DDP in culture medium during each assay) were called BGC‐823/DDP and MKN28/DDP. Cells were treated with DMSO (Sigma‐Aldrich) as the control group. Cells were then detected with an optical microscope (Opto‐Edu, Beijing, China).

### 2.4 Quantitative real‐time PCR

Total RNA was isolated from cultured cells or xenografts with TRIzol reagents (Invitrogen, Carlsbad, CA, USA). First Strand cDNA synthesis kit (Takara, Otsu, Shiga, Japan) was employed to perform reverse transcription. The iCycler IQ multi‐color Detection System (Bio‐Rad, Hercules, CA, USA) with IQ SYBR Green Supermix (Bio‐Rad) were used for quantitative real‐time PCR (qRT–PCR). Data were calculated by the $2^{‐\Delta \Delta C_{t}}$ method. GAPDH or U6 served as the internal control. The primers were exhibited as below:

FOXD1‐AS1:
  forward: 5′‐TTTTAACGCCTGGACCTGAGAAT‐3′,
  reverse: 5′‐GTTAATAACGCTATGCTACAGCC‐3′;
FOXD1:
  forward: 5′‐GATCTGTGAGTTCATCAGCGGC‐3′,
  reverse: 5′‐TGACGAAGCAGTCGTTGAGCGA‐3′
miR‐466:
  forward: 5′‐CACTAGTGGTTCCGTTTAGTAG‐3′,
  reverse: 5′‐TTGTAGTCA CTAGGGCACC‐3′;
PIK3CA:
  forward: 5′‐CTCCACGACCATCATCAGG‐3′,
  reverse: 5′‐CCTCACGGAGGCATTCTAAAG‐3′;
U6 small nuclear RNA (U6):
  forward: 5′‐CTCGCTTCGGCAGCACA‐3′,
  reverse: 5′‐AACGCT TCACGAATTTGCGT‐3′;
GAPDH:
  forward: 5′‐AAGGTGAAGGTCGGAGTCA‐3′,
  reverse: 5′‐GGAAGATG GTGATGGGATTT‐3′.

### 2.5 Cell transfection

Cells were added to 6‐well plates when cell fusion was up to 50–80%. The specific short hairpin (sh)RNA against FOXD1‐AS1 (shFOXD1‐AS1#1 and shFOXD1‐AS1#2) and FOXD1 (shFOXD1) and their corresponding NC (shCtrl) were constructed by GeneChem (Shanghai, China). Simultaneously, pcDNA3.1 vector targeting FOXD1‐AS1 or FOXD1 and their respective NC (empty pcDNA3.1 vector) were also constructed by GeneChem. Construction and synthesis of microRNA (miR)‐466 mimics and miR‐NC were accomplished by GenePharma (Shanghai, China). The above‐mentioned transfections conducted by Lipofectamine 3000 (Thermo Fisher Scientific) were finished after 48 h.

### 2.6 CCK‐8 assay

The transfected MKN45 or AGS cells were seeded in 96‐well plates before CCK‐8 reagents (10 μL; Dojindo, Tokyo, Japan) were added. The absorbance at the wavelength of 450 nm was monitored by a microplate reader (Thermo Fisher Scientific).

### 2.7 EdU assay

Transfected cells were incubated for 2 h with 50 μm EdU (RiboBio, Guangdong, China), followed by staining with DAPI (Invitrogen). The images were then captured by fluorescence microscopy (Nikon, Tokyo, Japan), and EdU‐positive cells were visualized and counted. The ratio of the total number of EdU‐positive cells to the total number of DAPI chromogenic cells was taken as the EdU‐positive rate.

### 2.8 TUNEL assay

Cells were transfected and rinsed with PBS (Thermo Fisher Scientific), followed by fixation in 1% paraformaldehyde (PFA; Sigma‐Aldrich). The apoptotic cells were stained with TUNEL solution (Roche, Mannheim, Germany). After the treatment with DAPI, fluorescence microscopy was applied to capture the images.

### 2.9 Transwell migration and invasion assays

Cell migration and invasion assay were carried out with Transwell chambers (8 μm; Costar, Boston, MA, USA). Transfected cells in 24‐well plates at a concentration of 5 × 10^4^ per well were added into the upper chambers, and medium containing 10% FBS was placed in the bottom chambers. Transwell chambers for invasion assay were coated with Matrigel (BD Biosciences, San Jose, CA, USA). After 24 h, migratory or invasive cells were fixed in 70% methanol (Sigma‐Aldrich) and then stained with 0.1% crystal violet (Sigma‐Aldrich). Cells were counted with the fluorescence microscopy.

### 2.10 Western blot

RIPA lysis buffer (Beyotime) was utilized to lyse cultured cells. Then, proteins were isolated by SDS/PAGE gels (Bio‐Rad) and shifted onto PVDF membranes (Millipore, Billerica, MA, USA). Primary antibodies acquired from Abcam (Cambridge, MA, USA) were added to PVDF membranes and incubated together at 4 °C overnight. After that, secondary antibodies were added. Immunoblots were detected using ECL Reagents (Pierce) and then subjected to image j software (NIH, Bethesda, MD, USA).

### 2.11 RNA pull‐down assay

A Pierce (Louisville, KY, USA) Magnetic RNA‐Protein Pull‐Down Kit bought from Thermo Fisher Scientific was used to conduct RNA pull‐down assay. The cells were lysed and then lysates were cultivated with the biotinylated RNA (Bio‐miR‐466 or Bio‐NC) as well as magnetic beads at 4 °C for 1 h. Finally, the precipitated RNA were detected by qRT–PCR.

### 2.12 RNA immunoprecipitation assay

Magna RIPTM RNA kit (Millipore) was utilized to conduct RNA immunoprecipitation (RIP) assay in line with the supplier’s protocol. Transfected MKN45 or AGS cells at a confluence of 80–90% were lysed with RIPA plus protease inhibitor and RNA enzyme inhibitor. IgG, Ago2, and eIF4G antibodies (Abcam) were separately added and incubated with cell extraction. After digesting proteins, immunoprecipitated RNA was collected and subjected to qRT–PCR after purification.

### 2.13 Co‐immunoprecipitation (Co‐IP) assay

The protein lysates were extracted from cultured MKN45 and AGS cells and quantitated. The interacting proteins were co‐precipitated using the specific antibodies (Abcam), followed by SDS/PAGE. Finally, immunocomplex was analyzed with western blotting.

### 2.14 Subcellular fractionation

The isolation of nuclear and cytoplasmic fractions was accomplished by the Nuclear/Cytosol Fractionation Kit (Biovision, San Francisco Bay, CA, USA). Quantitative Real‐time PCR was then carried out to evaluate the expression pattern of FOXD1‐AS1, GAPDH, and U6 in nuclear and cytoplasm fractions.

### 2.15 Luciferase reporter assay

FOXD1‐AS1‐WT/MUT and FOXD1‐WT/MUT were established by GenePharma and sub‐cloned into the pmirGLO vector (GeneChem). FOXD1‐AS1‐WT/MUT or FOXD1‐WT/MUT was transfected into HKT‐293T cells with indicated transfection plasmids. Luciferase activity was tested by applying Dual‐Luciferase Reporter Assay Kit (Promega, Madison, WI, USA).

### 2.16 *In vivo* growth and metastasis experiments

BALB/c‐nude mice (4–5 weeks of age) from Shi Laike Company (Shanghai, China) were maintained in a specific pathogen‐free environment with air‐conditioning for subsequent use. All procedures regarding animals were approved by the Animal Welfare and Research Ethics Committee at Tongren Hospital, Shanghai Jiao Tong University School of Medicine and conducted in line with the guideline of the Institutional Animal Care and Use Committee of our institution. For *in vivo* growth assay, AGS cells transfected with shFOXD1‐AS1 or control were injected subcutaneously into the BALB/c‐nude mice. Tumor size was recorded every 4 days and volumes were evaluated by length and width. The mice were sacrificed 4 weeks later, and the tumors were resected and weighed. The expression levels of indicated genes in these tumors were analyzed via *in situ* hybridization (ISH) or immunohistochemical (IHC) staining in the light of previous protocols (Chen *et al*., 2017). With respect to the *in vivo* metastatic model, FOXD1‐AS1‐silenced AGS cells and corresponding control cells were injected intravenously into mice through the tail vein. Two months later, mice were sacrificed and the number of metastatic nodules in excised liver or lung was counted by dissecting microscope after hematoxylin and eosin (H&E; Sigma‐Aldrich) staining.

### 2.17 Statistical analysis

Assays in this research were carried out at least three times. The spss 19.0 (SPSS, Chicago, IL, USA) was used to perform statistical analysis. Data were all expressed as mean ± SD. Student’s *t*‐test or one‐way/two‐way ANOVA was applied to analyze significant differences among groups. *P* < 0.05 was denoted to be statistically significant.

## 3 Results

### 3.1 FOXD1‐AS1 upregulation contributes to the malignant phenotypes in GC

**Fig. 1:**
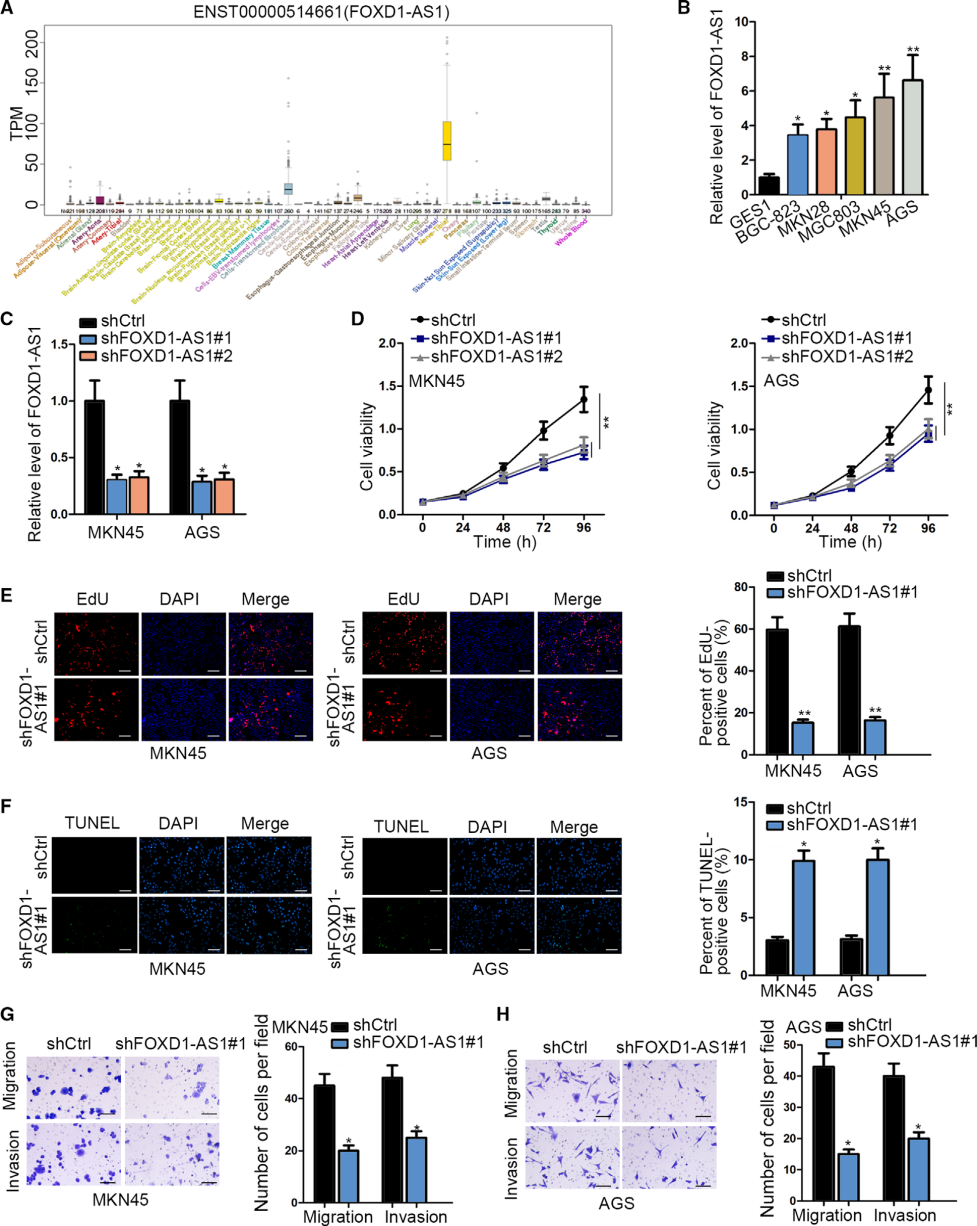
FOXD1‐AS1 was upregulated in GC cell lines and its inhibition restrained GC cell proliferation and motility. (A) UCSC indicated that FOXD1‐AS1 was underexpressed in normal human stomach tissues. (B) Relative expression level of FOXD1‐AS1 in GC cell lines; GES1 cell line was determined by qRT–PCR (*n* = 6, one‐way ANOVA). (C) qRT–PCR result of FOXD1‐AS1 in MKN45 and AGS cells under the transfection of shCtrl or two shRNA against FOXD1‐AS1. (*n* = 6, one‐way ANOVA). (D) The viability in MKN45 and AGS cells was evaluated by CCK‐8 assay. (*n* = 6, two‐way ANOVA). (E,F) Cell proliferation and apoptosis in MKN45 and AGS cells with or without FOXD1‐AS1 inhibition were examined via EdU and TUNEL assays, respectively (*n* = 6, Student’s *t*‐test, scale bar: 200 μm). (G,H) Transwell assay was conducted to analyze the migration and invasion in these two GC cells under FOXD1‐AS1 suppression. (*n* = 6, Student’s *t*‐test, scale bar: 100 μm). Data are shown as mean ± SD (standard deviation). Error bars indicate SD. *P < 0.05, **P < 0.01.

To probe the biological role of FOXD1‐AS1 in GC progression, we first evaluated its expression pattern in normal and diseased conditions. As revealed by UCSC, the expression of FOXD1‐AS1 was low in human normal stomach tissues (Fig. 1A). However, FOXD1‐AS1 showed a high expression level in GC cell lines compared with the normal GES1 cells, and MKN45 and AGS cells presented the higher FOXD1‐AS1 level among the five GC cell lines (Fig. 1B).

Based on these results, loss‐of‐function assays were conducted in MKN45 and AGS cells to analyze the function of FOXD1‐AS1 in GC. The qRT–PCR revealed that FOXD1‐AS1 was definitely downregulated in MKN45 and AGS cells transfected with shFOXD1‐AS1, and cell viability was suppressed after FOXD1‐AS1 was silenced (Fig. 1C,D). Short hairpin FOXD1‐AS1#1 was selected to continue our study because of the optimal transfection efficiency. Moreover, we found that silenced FOXD1‐AS1 obviously hindered the proliferation but induced the apoptosis in MKN45 and AGS cells (Fig. 1E,F). Furthermore, the migratory and invasive abilities of MKN45 and AGS cells were repressed upon FOXD1‐AS1 inhibition (Fig. 1G,H). Taken together, these results revealed that FOXD1‐AS1 is upregulated and plays a tumorigenic role in GC.

### 3.2 FOXD1‐AS1 triggers cisplatin resistance of GC cells

**Fig. 2:**
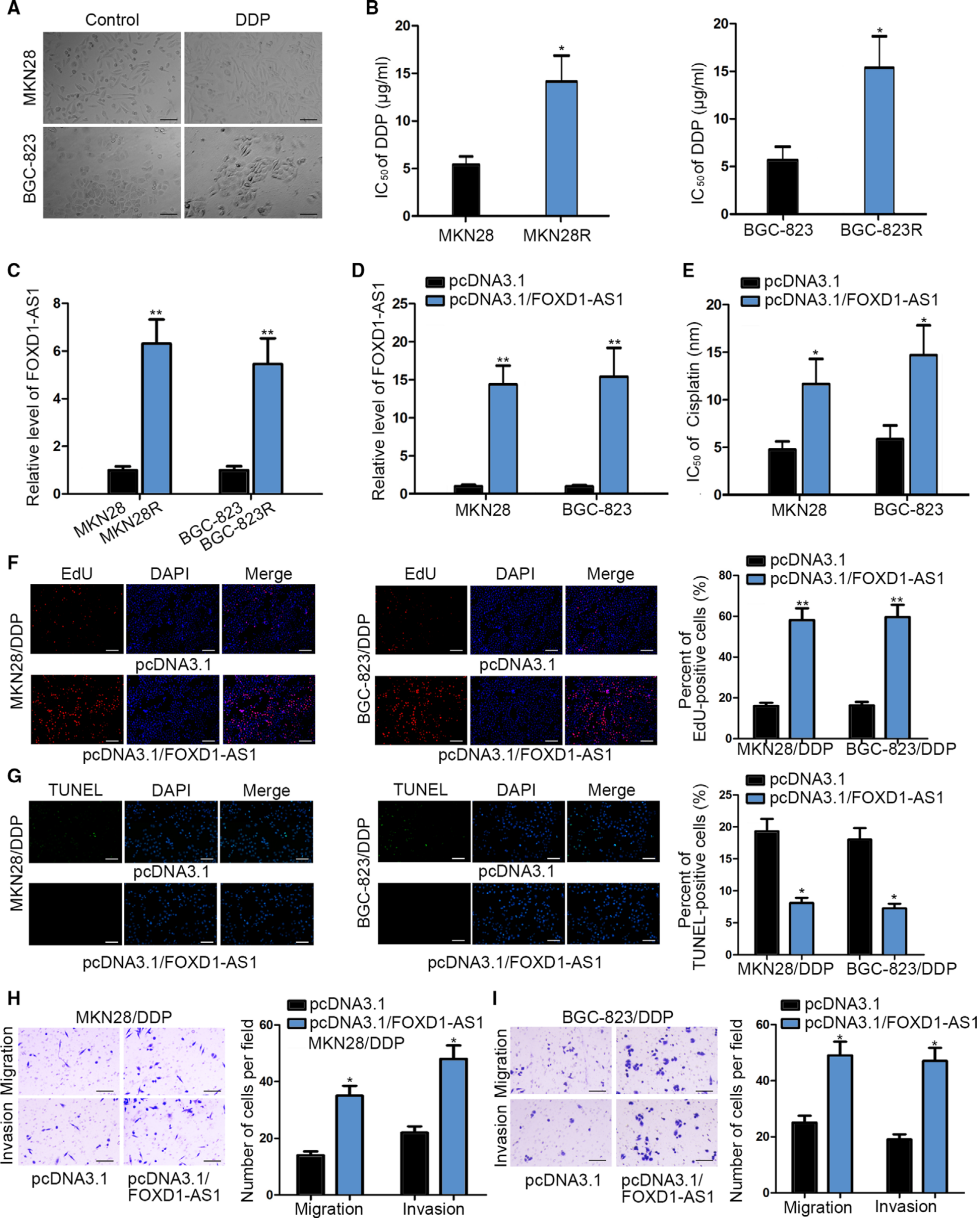
FOXD1‐AS1 upregulation contributed to the resistance of GC cells to DDP. (A) Morphological images of MKN28 and BGC‐823 cells and MKN28R and BGC‐823R cells that underwent continuous DDP treatment (*n* = 3, scale bar: 200 μm). (B) IC_50_ value of MKN28 and BGC‐823 cells responding to DDP treatment was determined by CCK‐8 assay. (C) qRT–PCR result of FOXD1‐AS1 level in MKN28R and BGC‐823R cells and the parental cells. (D) qRT–PCR analysis of FOXD1‐AS1 expression in MKN28 and BGC‐823 cells transfected with pcDNA3.1 or pcDNA3.1/FOXD1‐AS1. (E) CCK‐8 assay indicated an increased IC_50_ value in FOXD1‐AS1‐overexpressed MKN28 and BGC‐823 cells. (F,G) Cell proliferative ability and apoptotic rate in DDP‐treated MKN28 and BGC‐823 cells with or without FOXD1‐AS1 upregulation were evaluated using EdU and TUNEL assays (*n* = 6, scale bar: 200 μm). (H,I) The migratory and invasive abilities of indicated cells were assessed by Transwell assays (*n* = 6, scale bar: 200 μm). Data are shown as mean ± SD (standard deviation). Error bars indicate SD. *P < 0.05, **P < 0.01.

Since chemoresistance is identified as a major obstacle for GC treatment, we investigated the effect of FOXD1‐AS1 on the resistance of GC cells to cisplatin (DDP), a commonly used chemotherapeutic drug. As indicated in Fig. 2A, we discovered that GC cells changed into a spindly shape with fewer intercellular connections after long‐term DDP treatment. This suggested the potential of DDP resistance in these cells, which were subsequently named MKN28R and BGC‐823R cells. Compared with parental MKN28 and BGC‐823 cells, MKN28R and BGC‐823R cells presented higher IC_50_ values (Fig. 2B), indicating that the DDP resistance of MKN28R and BGC‐823R cells was strengthened. Meanwhile, we also discovered that the expression of FOXD1‐AS1 was dramatically upregulated in MKN28R and BGC‐823R cells relative to that in the parental controls (Fig. 2C). Therefore, we suspected that FOXD1‐AS1 might contribute to DDP resistance in GC.

To probe the function of FOXD1‐AS1 on GC cell DDP resistance, we conducted gain‐of‐function experiments. As a result, we discovered that the IC_50_ value of MKN28 and BGC‐823 cells was markedly heightened with the upregulation of FOXD1‐AS1 (Fig. 2D,E), implying that FOXD1‐AS1 overexpression enhanced DDP resistance in GC. Moreover, it was revealed that overexpression of FOXD1‐AS1 promoted the proliferation and inhibited the apoptosis in MKN28 and BGC‐823 cells treated with DDP (Fig. 2F,G). In addition, the migration and invasion of DDP‐treated MKN28 and BGC‐823 cells was significantly improved by FOXD1‐AS1 upregulation (Fig. 2H,I). Likewise, overexpressed FOXD1‐AS1 led to elevated DDP resistance in MKN45 and AGS cells (Fig. S1A,B). To sum up, FOXD1‐AS1 promotes the resistance of GC cells to DDP treatment.

### 3.3 FOXD1‐AS1 regulates FOXD1 expression at translational level

**Fig. 3:**
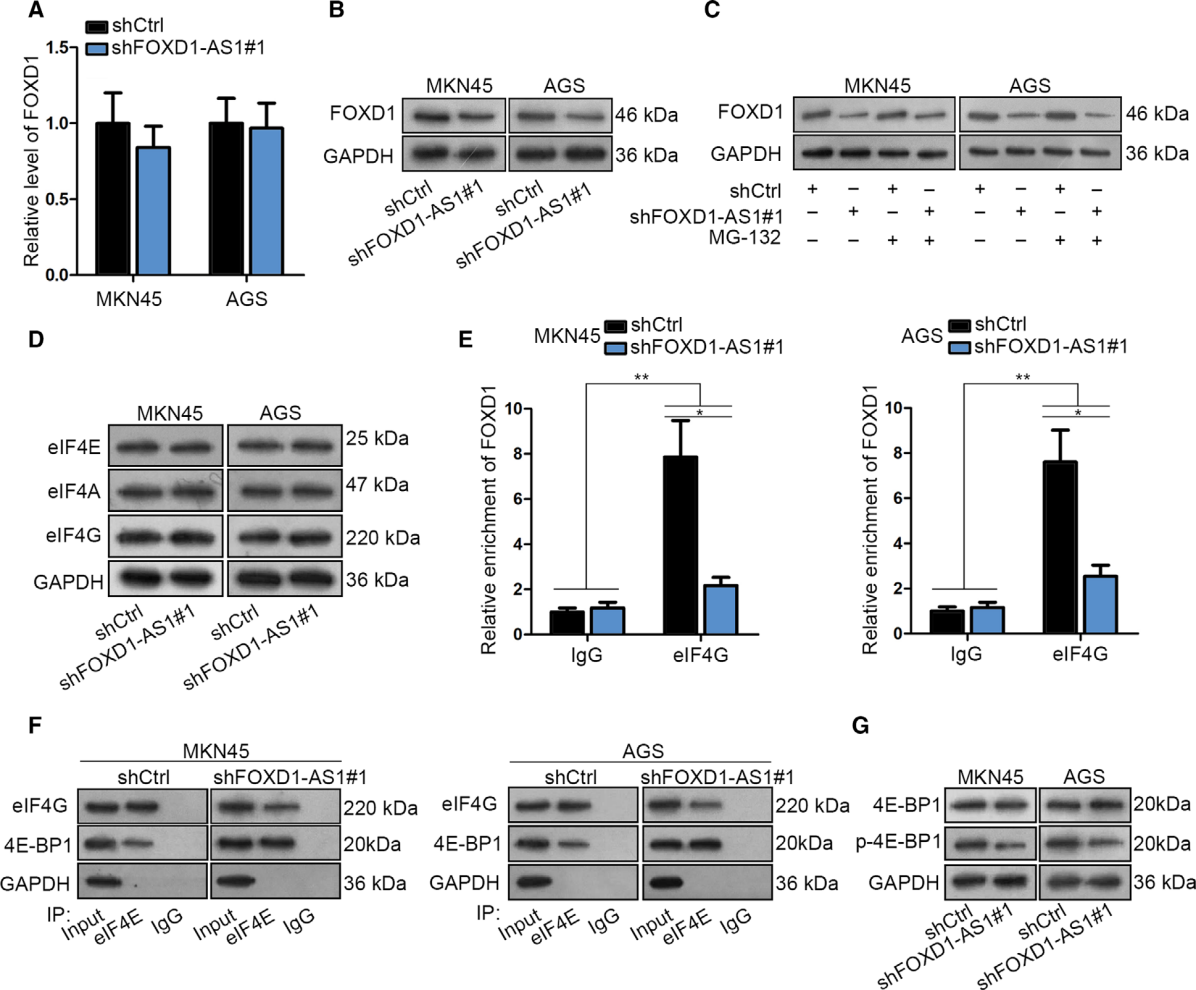
FOXD1‐AS1 facilitated FOXD1 mRNA translation in GC by affecting the assembly of eIF4E/eIF4G translational complex. (A,B) The impact of FOXD1‐AS1 on the mRNA and protein levels of FOXD1 was tested by qRT–PCR and western blot, respectively (*n* = 3, Student’s *t*‐test). (C) The level of FOXD1 protein in indicated cells was detected via western blotting. (*n* = 3). (D) Western blot analysis of the protein levels of eIF4E, eIF4G and 4E‐BP1 in MKN45 and AGS cells with or without FOXD1‐AS1 depletion (*n* = 3). (E) The interaction between eIF4G and FOXD1 mRNA in MKN45 and AGS cells was determined by RIP assay (*n* = 3, Student’s *t*‐test and one‐way ANOVA). (F) The interaction of eIF4E with eIF4G or 4E‐BP1 in shCtrl‐ or shFOXD1‐AS1#1‐transfected GC cells was analyzed by Co‐IP assay. (G) The level of 4E‐BP1 and p‐4E‐BP1 in indicated cells was evaluated by western blot (*n* = 3). Data are shown as mean ± SD (standard deviation). Error bars indicate SD. *P < 0.05, **P < 0.01.

Next, we sought to explain the detailed mechanism whereby FOXD1‐AS1 affected GC progression. At this point, forkhead box D1 (FOXD1), the cognate sense transcript of FOXD1‐AS1, attracted our attention because it has been verified to be upregulated in gastric adenocarcinoma tissues (Xu *et al*., 2016) and is recognized as an oncogene in several cancers (Li *et al*., 2018). Intriguingly, we observed an evident inhibition of silenced FOXD1‐AS1 on the expression of FOXD1 at protein level but not at mRNA level (Fig. 3A,B), suggesting that FOXD1‐AS1 might affect FOXD1 expression in GC at translational or post‐translational levels. Therefore, we used MG132, a proteasome inhibitor, to detect FOXD1 protein level in shCtrl‐ or shFOXD1‐AS1#1‐transfected GC cells. Consequently, despite the application of MG132, FOXD1 protein level was still reduced by FOXD1‐AS1 depletion in MKN45 and AGS cells (Fig. 3C), proving that FOXD1‐AS1 regulated FOXD1 expression at translational level in GC. In this regard, we focused on the effect of FOXD1‐AS1 on the assembly of eukaryotic translation initiation complex (eIF4G‐eIF4E‐eIF4A). Interestingly, it was demonstrated that knockdown of FOXD1‐AS1 had no apparent influence on the expression of eIF4G, eIF4E or eIF4A (Fig. 3D). However, FOXD1‐AS1 knockdown obstructed the binding of FOXD1 mRNA to eIF4G, the core protein of the aforementioned eukaryotic translation initiation complex, in two GC cells (Fig. 3E). More importantly, silencing of FOXD1‐AS1 inhibited the interaction between eIF4E and eIF4G but enhanced the interaction of eIF4E with eukaryotic initiation factor 4E‐binding protein 1 (4E‐BP1) (Fig. 3F). It is well known that the hypophosphorylated form of 4E‐BP1 competes with eIF4G to bind with eIF4E and finally leads to repressed translation. Hence, we speculated that FOXD1‐AS1 might regulate the phosphorylation of 4E‐BP1 to affect eIF4E‐4E‐BP1 interaction. As anticipated, silencing FOXD1‐AS1 reduced the level of p‐4E‐BP1 in both MKN45 and AGS cells (Fig. 3G). Hence, we concluded that FOXD1‐AS1 facilitates FOXD1 translation through strengthening eIF4G‐eIF4E interaction via phosphorylation of 4E‐BP1.

### 3.4 FOXD1‐AS1 drives the phosphorylation of 4E‐BP1 by activating PI3K/AKT/mTOR pathway

**Fig. 4:**
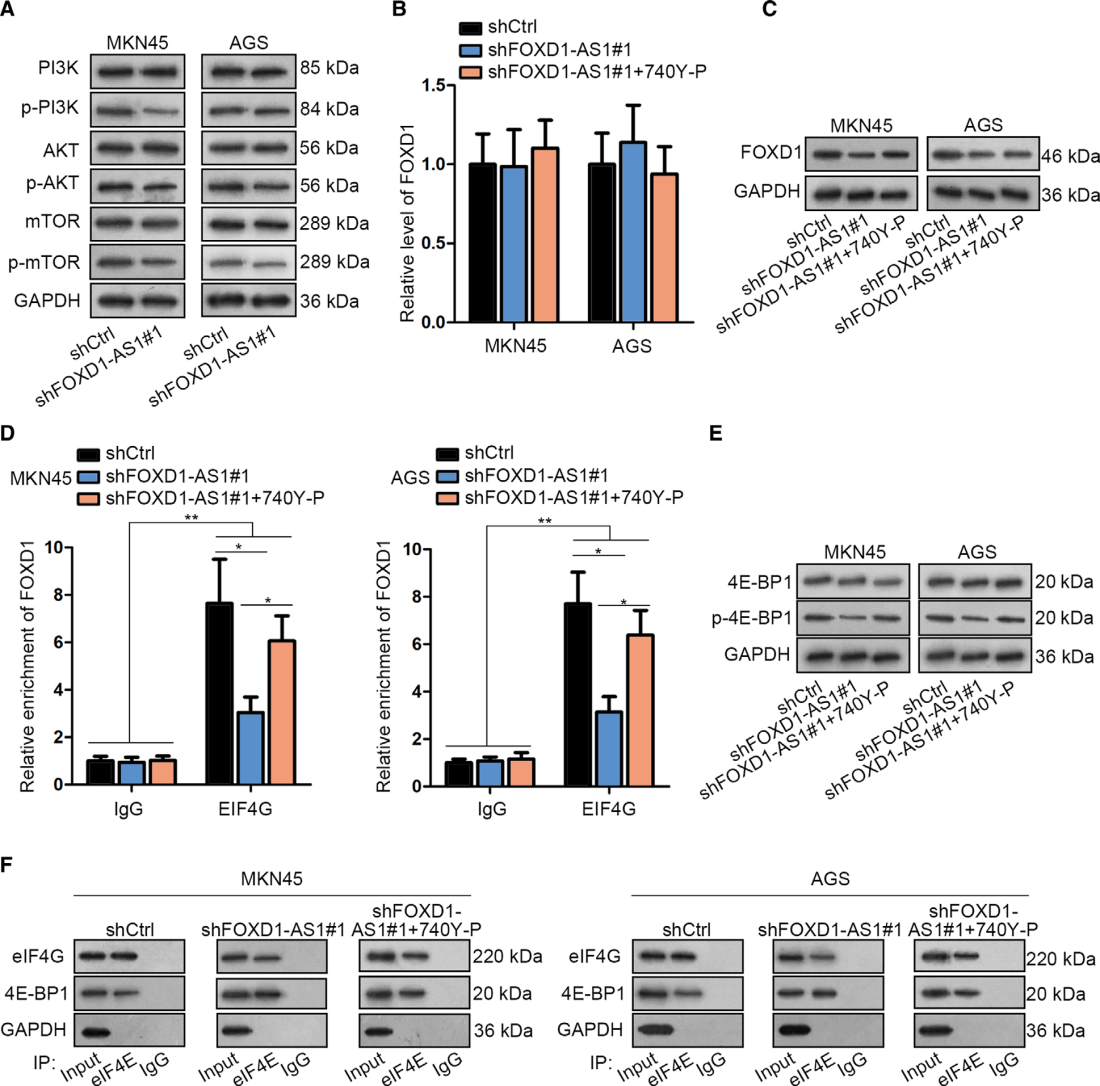
FOXD1‐AS1 enhanced 4E‐BP1 phosphorylation by activation of the PI3K/AKT/mTOR pathway. (A) The influence of FOXD1‐AS1 knockdown on the level of proteins associated with PI3K/AKT/mTOR signaling was determined via western blot (*n* = 3). (B,C) FOXD1 mRNA and protein levels in MKN45 and AGS cells with FOXD1‐AS1 silencing or together with 740Y‐P treatment was assessed by qRT–PCR and western blot (*n* = 3, one‐way ANOVA). (D) RIP assay was carried out to estimate the interaction between eIF4G and FOXD1 mRNA in MKN45 and AGS cells (*n* = 5, one‐way ANOVA). (E) The phosphorylation of 4E‐BP1 in indicated cells was analyzed by western blot (*n* = 5). (F) Co‐IP assay was applied to assess the interaction between eIF4E and eIF4G or 4E‐BP1 in indicated GC cells (*n* = 5). Data are shown as mean ± SD (standard deviation). Error bars indicate SD. *P < 0.05, **P < 0.01.

4E‐BP1 is known as the downstream gene of PI3K/AKT/mTOR signaling pathway, and its phosphorylation can be enhanced via this pathway. In this regard, we attempted to investigate whether FOXD1‐AS1 regulated the PI3K/AKT/mTOR signaling pathway in GC. Consequently, we found that FOXD1‐AS1 depletion decreased the phosphorylated levels, rather than the total levels, of PI3K, AKT, and mTOR in both MKN45 and AGS cells (Fig. 4A), suggesting the positive regulation of FOXD1‐AS1 in this pathway. Further, to confirm whether FOXD1‐AS1 modulated FOXD1 in GC via the PI3K/AKT/mTOR pathway, PI3K activator (740Y‐P) was used to conduct rescue assays in FOXD1‐AS1‐inhibited GC cells. As a result, 740Y‐P did not affect the FOXD1 mRNA level but counteracted the inhibition of the FOXD1 protein level by FOXD1‐AS1 knockdown (Fig. 4B,C). Moreover, RIP assay validated that repressed binding of FOXD1 with eIF4E in FOXD1‐AS1‐silenced cells was restored by 740Y‐P (Fig. 4D). Subsequently, we validated that 740Y‐P recovered the phosphorylation of 4E‐BP1 and the interaction of eIF4E with eIF4G in shFOXD1‐AS1#1‐transfected cells (Fig. 4E,F). Collectively, FOXD1‐AS1 activates the PI3K/AKT/mTOR pathway so as to increase FOXD1 expression in GC via stimulation of eIF4E‐eIF4G interaction.

### 3.5 FOXD1‐AS1 regulates the PI3K/AKT/mTOR pathway by acting as a competing endogenous (ce)RNA of PIK3CA by absorption of miR‐466

**Fig. 5:**
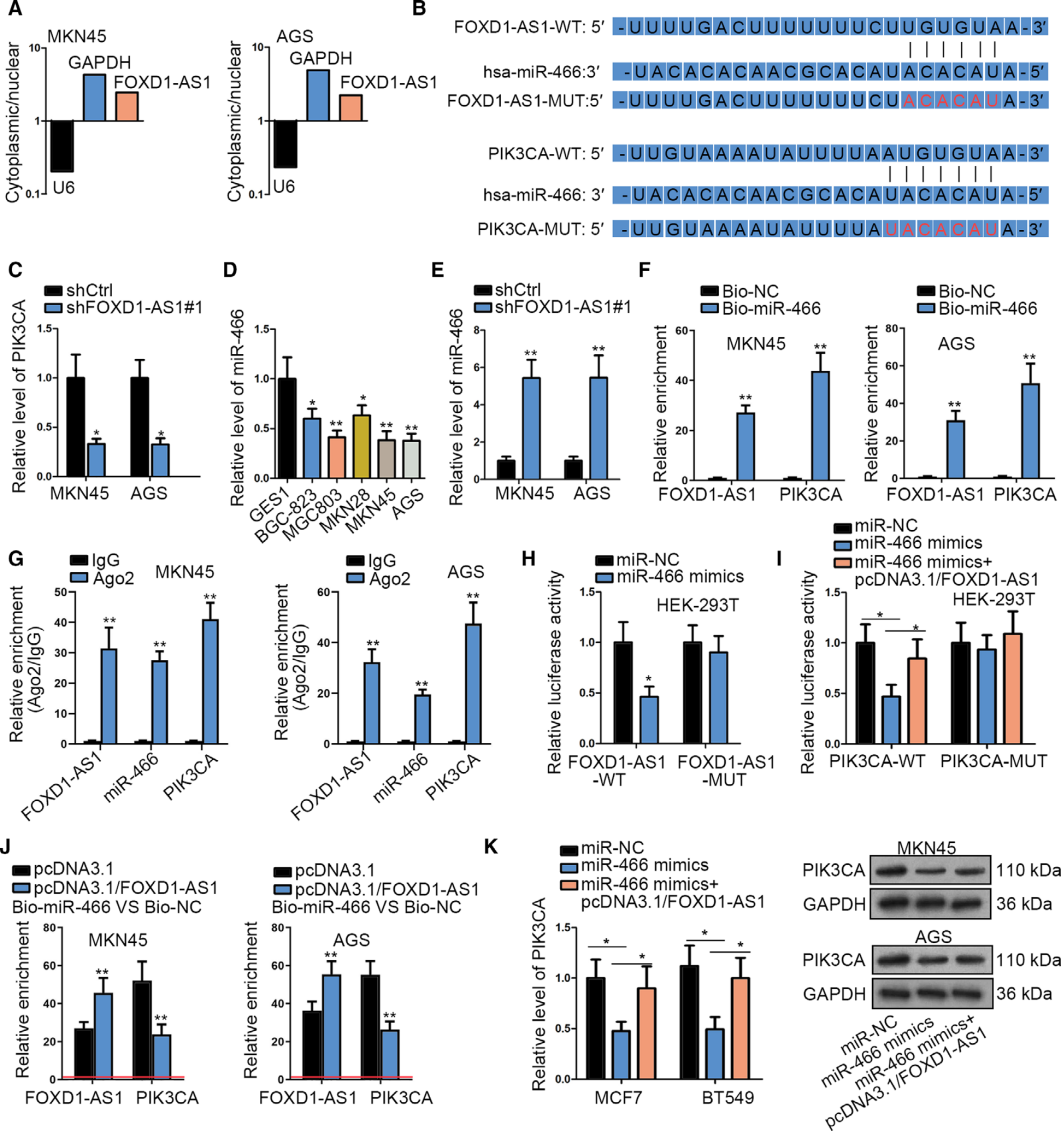
FOXD1‐AS1 was mainly located in the cytoplasm and functioned as a ceRNA of PIK3CA by interacting with miR‐466. (A) Subcellular fractionation followed by qRT–PCR was conducted to determine the subcellular distribution of FOXD1‐AS1 in GC cells (*n* = 4). (B) Sequences used to construct luciferase reporter plasmids. (C) qRT–PCR result of PIK3CA expression in FOXD1‐AS1‐silenced MKN45 and AGS cells (*n* = 5, Student’s *t*‐test). (D) qRT–PCR result of miR‐466 level in GC cell lines and GES1 cell line (*n* = 5, one‐way ANOVA). (E) qRT–PCR result of miR‐466 expression in MKN45 and AGS cells with or without FOXD1‐AS1 silencing (*n* = 5, Student’s *t*‐test). (F,G) The interaction of miR‐466 with PIK3CA and FOXD1‐AS1 was evaluated by RNA pull‐down (F) and RIP assays (G) (*n* = 5, Student’s *t*‐test). (H,I) Luciferase reporter assays proved that there was a competition between FOXD1‐AS1 and PIK3CA mRNA in binding with miR‐466 (*n* = 5, Student’s *t*‐test and one‐way ANOVA). (J) RNA pull‐down assay revealed the competition between FOXD1‐AS1 and PIK3CA in binding to miR‐466 (*n* = 5, Student’s *t*‐test). (K) The mRNA and protein expressions of PIK3CA in GC cells transfected with indicated plasmids were assessed by qRT–PCR and western blot (*n* = 5, one‐way ANOVA). Data are shown as mean ± SD (standard deviation). Error bars indicate SD. *P < 0.05, **P < 0.01.

We wanted to explore in depth how FOXD1‐AS1 regulated PI3K/AKT/mTOR signaling in GC. Considering that the function that lncRNA exerted was dependent on their cellular localization (Sun and Kraus, 2015), we first detected the distribution of FOXD1‐AS1 in GC cells. The results showed that FOXD1‐AS1 were mainly located in the cytoplasm of MKN45 and AGS cells (Fig. 5A), indicating that FOXD1‐AS1 might serve as a ceRNA in GC. Fortunately, we discovered miR‐466 as the shared miRNA between FOXD1‐AS1 and PIK3CA, a PI3K alpha subunit that is involved in the activation of PI3K signaling cascades (Fig. 5B). We also discovered that the PIK3CA level was sharply reduced upon FOXD1‐AS1 inhibition (Fig. 5C), which suggested a regulatory role of FOXD1‐AS1 in PIK3CA expression. Meanwhile, miR‐466, a recently recognized tumor suppressor in several human cancers (Colden *et al*., 2017), was proved to be expressed at a low level in GC cell lines versus the normal GES‐1 cells (Fig. 5D). Also, the expression of miR‐466 was pronouncedly upregulated in FOXD1‐AS1‐downregulated GC cells (Fig. 5E). Moreover, it was confirmed that both FOXD1‐AS1 and PIK3CA were highly enriched in Bio‐miR‐466 groups (Fig. 5F), indicating the interactions of miR‐466 with both FOXD1‐AS1 and PIK3CA. Additionally, such interactions were proved to occur in miR‐466‐guided RNA‐induced silencing complexes (RISC) (Fig. 5G). Noticeably, the luciferase activities of FOXD1‐AS1‐WT and PIK3CA‐WT were both lessened by ectopic expression of miR‐466, whereas the repressing effect of miR‐466 on the luciferase activity of PIK3CA‐WT was offset by FOXD1‐AS1 upregulation (Fig. 5H,I). Interestingly, the above phenomenon was attributed to the reduced PIK3CA targeting by miR‐466, which resulted from enhanced binding of miR‐466 to FOXD1‐AS1 (Fig. 5J). Further, PIK3CA mRNA and protein levels were impaired by miR‐466 overexpression but reverted under FOXD1‐AS1 upregulation (Fig. 5K). Based on these results, FOXD1‐AS1 activates the PI3K/AKT/mTOR pathway through elevating PIK3CA expression via sponging miR‐466.

### 3.6 FOXD1 mediates the contribution of FOXD1‐AS1 to GC cell proliferation, motility, and DDP resistance

**Fig. 6:**
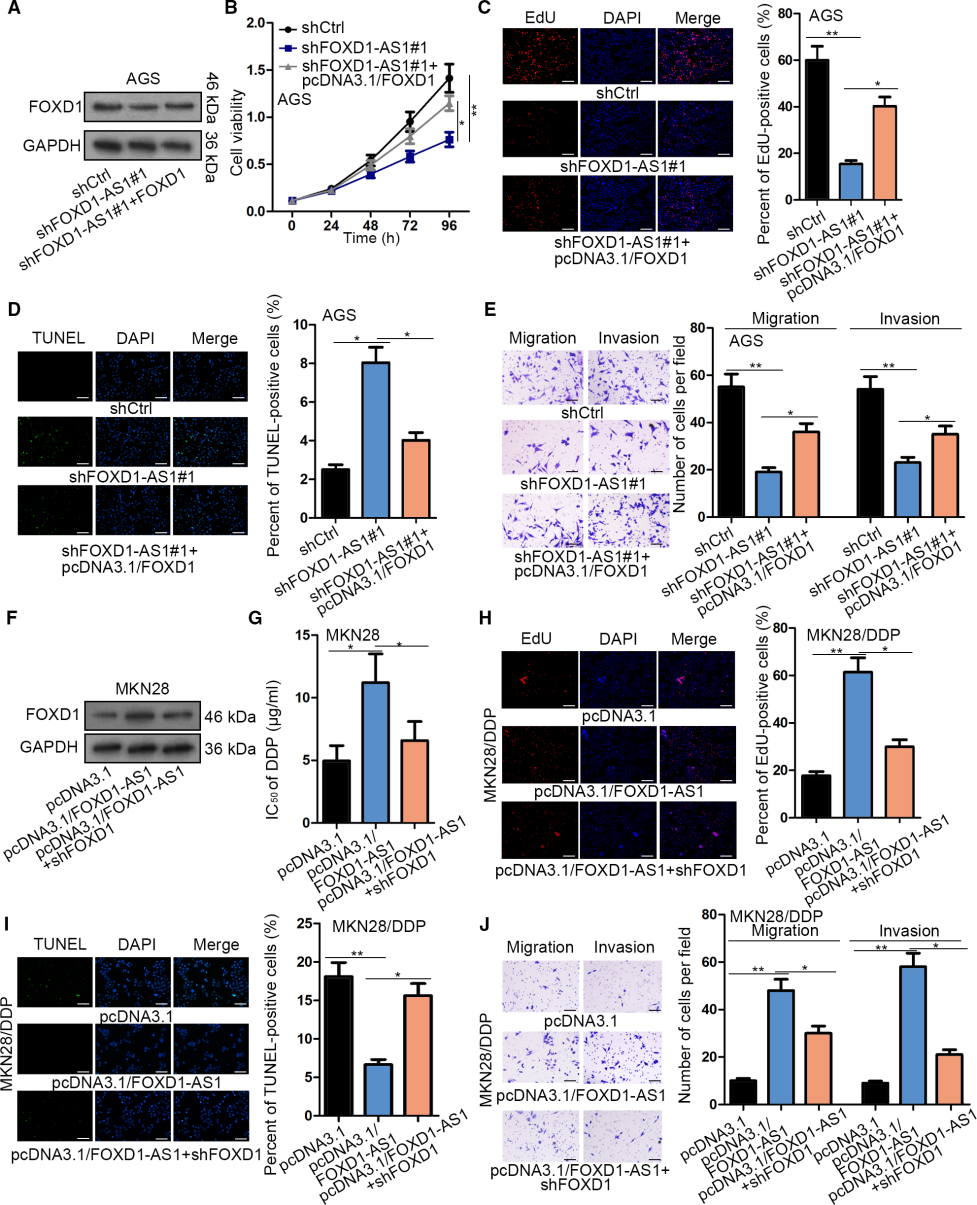
FOXD1 was responsible for FOXD1‐AS1‐affected GC progression and chemoresistance. (A) Western blot result of FOXD1 protein level in AGS cells transfected with shCtrl, shFOXD1‐AS1#1 or shFOXD1‐AS1#1 plus pcDNA3.1/FOXD1 (*n* = 3). (B–E) Cell viability, proliferation, apoptosis, and motility in AGS cells were detected by (B) CCK‐8 (*n* = 5, two‐way ANOVA); (C) EdU (*n* = 5, one‐way ANOVA) (scale bars: 200 μm); (D) TUNEL (*n* = 5, one‐way ANOVA; scale bar: 200 μm); and (E) Transwell assays (*n* = 5, one‐way ANOVA; scale bar: 200 μm). (F) Western blot was used to identify the level of FOXD1 protein in MKN28 cells transfected with pcDNA3.1, pcDNA3.1/FOXD1‐AS1 or pcDNA3.1/FOXD1‐AS1 plus shFOXD1 (*n* = 5). (G) The DDP resistance of MKN28 cells was estimated via CCK‐8 assay (*n* = 5, one‐way ANOVA). (H–J) The proliferation, apoptosis, and motility in DDP‐treated MKN28 cells under indicated transfections were evaluated through conducting (H) EdU (*n* = 5, one‐way ANOVA, scale bar: 200 μm), (I) TUNEL (*n* = 5, one‐way ANOVA, scale bar: 200 μm), and (J) Transwell assays (*n* = 5, one‐way ANOVA, scale bar: 200 μm). Data are shown as mean ± SD (standard deviation). Error bars indicate SD. *P < 0.05, **P < 0.01.

Next, we investigated whether FOXD1 participated in FOXD1‐AS1‐regulated GC biological processes. As displayed in Fig. 6A, co‐transfection of pcDNA3.1/FOXD1 rescued the protein level of FOXD1 reduced by FOXD1‐AS1 depletion. Subsequently, FOXD1 overexpression countervailed the suppressive effect of FOXD1‐AS1 silencing on the viability and proliferation of AGS cells (Fig. 6B,C). By contrast, the apoptosis increased by FOXD1‐AS1 knockdown was reversed by upregulation of FOXD1 (Fig. 6D). Moreover, cell migration and invasion inhibited by FOXD1‐AS1 downregulation were restored under FOXD1 overexpression (Fig. 6E). Thus, we concluded that FOXD1‐AS1 aggravates cell growth and migration in a FOXD1‐dependent manner in GC. We then assessed the role that the FOXD1‐AS1/FOXD1 axis played in the regulation of DDP resistance. As expected, DDP resistance of GC cells was strengthened under upregulated FOXD1 but declined upon FOXD1 downregulation (Fig. S1C–F), revealing that FOXD1 improved the resistance of GC cells to DDP. As a result, FOXD1 downregulation lowered the IC_50_ value that was augmented by FOXD1‐AS1 upregulation in MKN28 cells (Fig. 6F,G). Further, we showed evidence that FOXD1 depletion rescued the increase of proliferation, decrease of apoptosis, and induction of migration and invasion in MKN28 cells caused by FOXD1‐AS1 overexpression (Fig. 6H–J). The data above revealed that FOXD1‐AS1 enhances the growth and DDP resistance of GC cells by targeting FOXD1.

### 3.7 Knockdown of FOXD1‐AS1 impedes tumor growth and metastasis *in vivo*

**Fig. 7:**
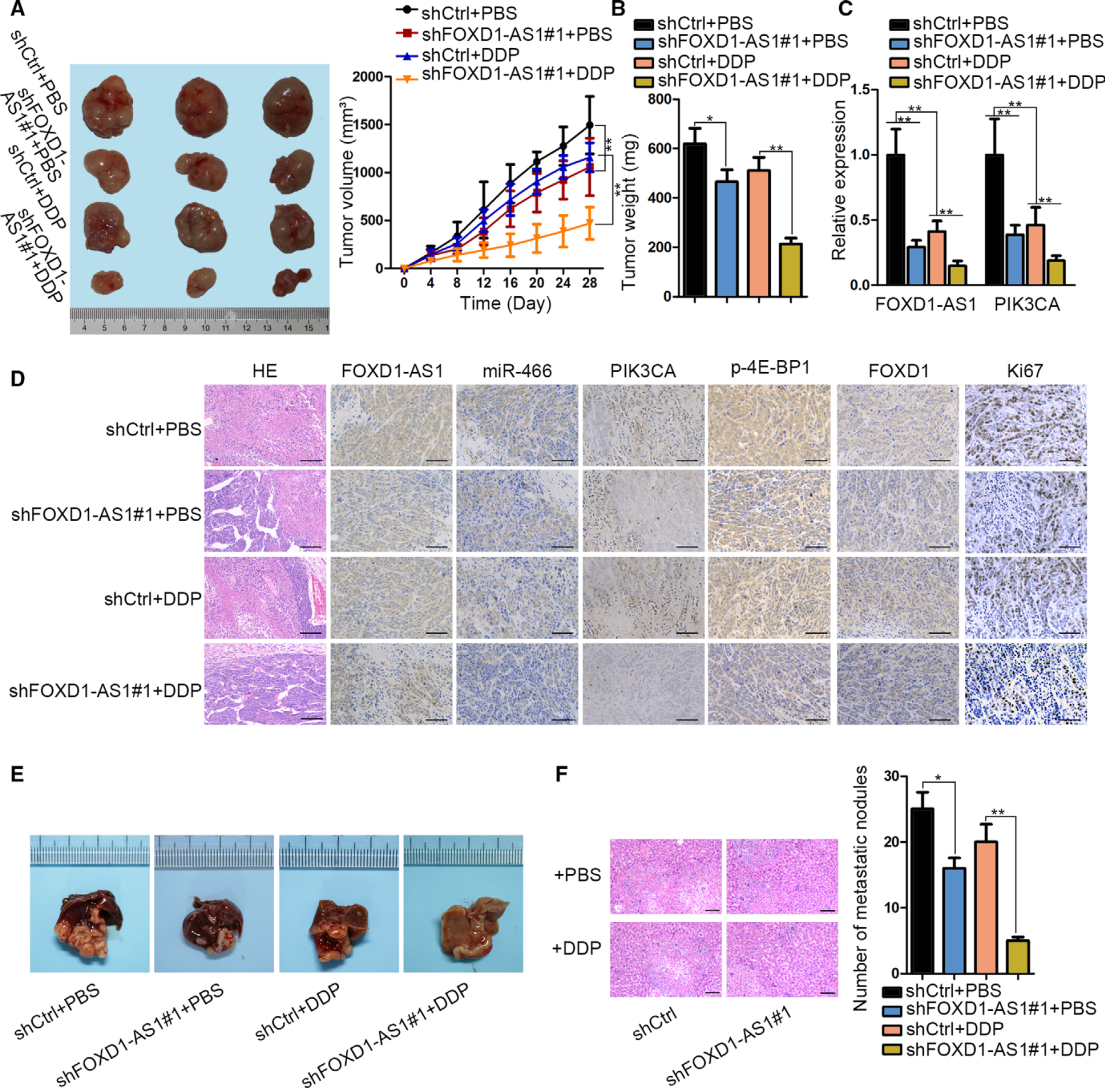
Depletion of FOXD1‐AS1 repressed the growth, metastasis, and DDP resistance of GC cells *in vivo*. (A,B) Representative images, volume and weight of tumors derived from mice with shCtrl‐ or shFOXD1‐AS1#1‐transfected AGS cells and together with PBS or DDP treatment (*n* = 3, one‐way/two‐way ANOVA). (C) qRT–PCR result of FOXD1‐AS1 and PIK3CA expressions in the tumors (*n* = 5, one‐way ANOVA). (D) ISH staining of FOXS1‐AS1 and miR‐466 as well as IHC staining of PIK3CA, p‐4E‐BP1, FOXD1, and Ki67 in tumors (*n* = 3, scale bars: 200 μm). (E) Representative images of livers with metastatic nodules from mice in indicated groups (*n* = 3). (F) HE staining of above livers and the quantitative diagram of metastatic nodules (*n* = 3, one‐way ANOVA). Data are shown as mean ± SD (standard deviation). Error bars indicate SD. *P < 0.05, **P < 0.01.

Finally, we aimed to certify our above findings using animal models. N agreement with our results, tumors from mice treated with PBS were smaller and lighter in the FOXD1‐AS1‐silenced group than those in the control group, revealing the promotional role of FOXD1‐AS1 in GC tumorigenesis. More importantly, results revealed that the tumor volume and weight from mice treated with DDP were decreased in FOXD1‐AS1‐inhibited AGS cells relative to shCtrl‐transfected AGS cells, suggesting that tumors with low FOXD1‐AS1 level were more sensitive to DDP treatment than that in shCtrl+DDP groups (Fig. 7A,B). In addition, we observed that the expression levels of both FOXD1‐AS1 and PIK3CA were remarkably decreased in tumors with FOXD1‐AS1‐depleted AGS cells, and their levels were further decreased upon DDP treatment (Fig. 7C). Moreover, it was shown that the staining of FOXD1‐AS1, PIK3CA, p‐4E‐BP1, FOXD1, and Ki67 exhibited a similar trend, whereas that of miR‐466 presented the opposite trend in tumors after FOXD1‐AS1 downregulation from mice with PBS or DDP treatment (Fig. 7D). Furthermore, *in vivo* metastatic assays showed that FOXD1‐AS1 knockdown decreased the number of metastatic nodules in both liver and lung, and the decrease was more significant with the treatment of DDP (Figs 7E,F and S2A,B). In conclusion, FOXD1‐AS1 accelerates tumor growth, metastasis, and chemoresistance in GC.

## 4 Discussion

In the current study, we revealed that FOXD1‐AS1 was an oncogenic lncRNA in the development of GC, as previously reported in glioma (Gao *et al*., 2020), which suggested FOXD1‐AS1 as a potential new target for GC treatment. FOXD1‐AS1 was also confirmed as a contributor to the resistance of GC cells to DDP, a commonly used chemotherapeutic drug for GC treatment (Liu *et al*., 2018b). In view of the unsatisfactory efficacy of chemotherapy due to the ultimately acquired chemoresistance of GC patients (Lei *et al*., 2013), our study might be helpful to improve the treatment effect of DDP for GC patients.

LncRNA have been reported to play multiple roles in cancers and affect cancer development by modulating gene expression (Cheetham *et al*., 2013). In terms of antisense lncRNA, they are usually confirmed to regulate the sense protein‐coding genes under various conditions. For example, SMN‐AS1 was discovered to affect the expression of its sense transcript SMN in spinal muscular atrophy (d’Ydewalle *et al*., 2017). Moreover, FOXC2‐AS1 was reported to enhance doxorubicin resistance in osteosarcoma by stimulating FOXC2 (Zhang *et al*., 2017). Similarly, FOXD1‐AS1 was validated to exert functions in GC relying on FOXD1, a transcription factor belonging to the forkhead family. FOXD1 was also recognized as a cancer‐promoter in various human malignancies, such as melanoma (Wu *et al*., 2018), lung cancer (Li *et al*., 2018), glioma (Cheng *et al*., 2016), colorectal cancer (Zhang *et al*., 2018), and breast cancer (Zhao *et al*., 2015).

Intriguingly, we discovered that FOXD1‐AS1 modulated FOXD1 expression in GC at protein level by affecting its translation. It has been revealed that the global translation rate of eukaryotic gene expression is mainly limited in the initiation step mediated by eIF4E (Hinnebusch, 2014) and that eIF4E is important for initiating the translation of pro‐oncogenic mRNA in cancers (2015). In this study, we observed no change in the expression levels of eIF4G, eIF4E or eIF4A, three core proteins in the eukaryotic translation initiation complex (eIF4G‐eIF4E‐eIF4A, also called the eIF4F complex). Interestingly, we further verified that FOXD1‐AS1 affected the assembly of the eIF4F complex (Kapp and Lorsch, 2004). It is known that eIF4E is indispensable for the recognition of 7‐methyl guanosine residue in the 5′ caps of all nuclear‐encoded eukaryotic mRNA (Richter and Sonenberg, 2005). And eIF4G is also important because it is a molecular scaffold with docking function for the assembly of the eIF4F complex (Jackson *et al*., 2010; Sonenberg and Hinnebusch, 2009). In this regard, the interaction of eIF4E with eIF4G is a prerequisite for the initiation of mRNA translation. However, 4E‐BP1, one of the downstream targets of mTOR (Hay and Sonenberg, 2004), can strongly bind with eIF4E via its hypophosphorylated form and thereby prevent the interaction between eIF4E and eIF4G (Musa *et al*., 2016). In the present study, we discovered that FOXD1‐AS1 enhanced the translation of FOXD1 protein in GC via hyperphosphorylation of 4E‐BP1 and therefore facilitated the formation of eIF4F complex.

4E‐BP1 is a well‐validated downstream target whose phosphorylation is modulated based on the activation of the PI3K/AKT/mTOR signaling pathway, a pathway that is frequently activated in numerous cancers, including GC (Fruman and Rommel, 2014; Tapia *et al*., 2014). The present study indicated that FOXD1‐AS1 might affect PI3K expression in GC cells. PIK3CA (phosphatidylinositol‐4,5‐bisphosphate 3‐kinase catalytic subunit alpha) is a subunit of PI3K protein that participates in PI3K/AKT/mTOR signaling, and its reduction leads to the suppression of this pathway (Wang *et al*., 2017). Moreover, PIK3CA has been recognized as a facilitator in GC by activation of the PI3K/AKT pathway (Liu *et al*., 2010). Herein, we identified that FOXD1‐AS1 is a cytoplasmic lncRNA in GC and it functions as a ceRNA of PIK3CA through competitive binding to miR‐466, a well‐known tumor suppressor in several cancers (Colden *et al*., 2017; Liu *et al*., 2018a; Tong *et al*., 2019).

**Fig. 8:**
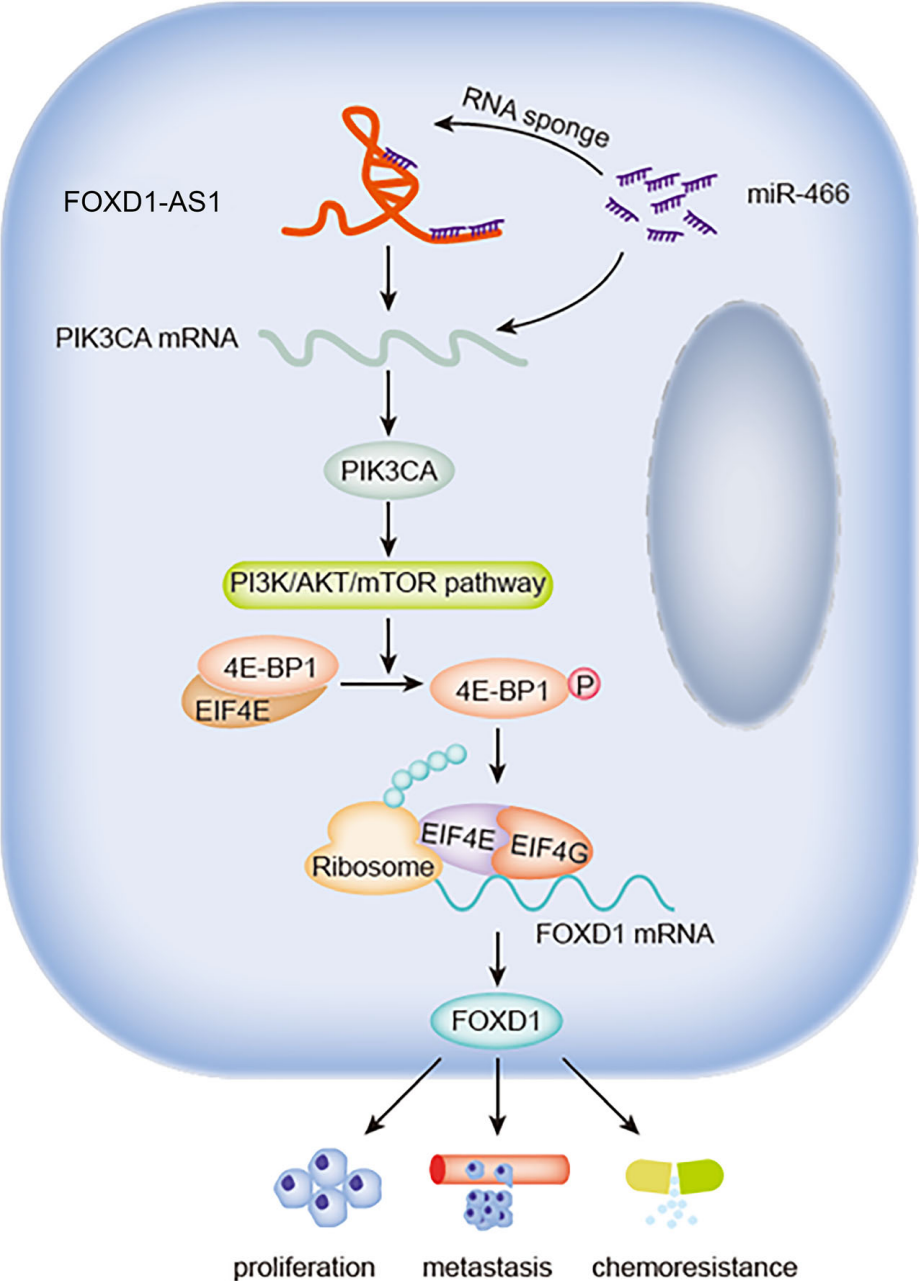
Graphical abstract illustrates the function and regulatory mechanism of FOXD1‐AS1 in AGS.

Our research elucidated that FOXD1‐AS1 released PIK3CA by sponging miR‐466 to activate PI3K/AKT/mTOR signaling and therefore hyperphosphorylating 4E‐BP1, thus promoting the interaction between eIF4E and eIF4G, and resulting in increased FOXD1 protein, and inevitably contributing to GC development and DDP resistance (Fig. 8). This study certified that FOXD1‐AS1 facilitated the progression and DDP resistance through regulation of FOXD1 protein translation. Our findings indicate FOXD1‐AS1 as a promising target for treating and even overcoming DDP resistance of GC patients. However, the clinical significance of FOXD1‐AS1 in GC needs to be explored further.

## Conflict of interest

The authors declare no conflict of interest.

## Author contributions

QW designed and conducted experiments, analyzed the statistical significance of data, and wrote the paper. JM and JW participated in designing and performing experiments. WM, YW, and MS helped to revise the manuscript.

## Supporting information


**Fig. S1.** Impact of FOXD1‐AS1 or FOXD1 on the resistance of GC cells to DDP treatment. (A) FOXD1‐AS1 expression in MKN45 and AGS cells transfected with pcDNA3.1 or pcDNA3.1/FOXD1‐AS1 was assayed via qRT–PCR (*n* = 5, Student’s *t*‐test. (B) The effect of FOXD1‐AS1 upregulation on the resistance of MKN45 and AGS cells to DDP treatment was detected by CCK‐8, EdU, TUNEL, and Transwell assays (*n* = 5, Student’s *t*‐test). (C,D) The levels of FOXD1 in FOXD1‐overexpressed AGS cells were determined by qRT–PCR and western blot (*n* = 5, Student’s *t*‐test) (C). Its impact on DDP‐resistant AGS cells was tested by CCK‐8, EdU, and Transwell assays (D) (*n* = 5, Student’s *t*‐test). (E,F) FOXD1 expression in FOXD1‐depleted MKN28R cells were determined by qRT–PCR and western blot (E). Its impact on DDP‐sensitivity of MKN28R cells was estimated via CCK‐8, EdU, TUNEL, and Transwell assays (F) (*n* = 5, Student’s *t*‐test). Data are shown as mean ± SD (standard deviation). Error bars indicate SD. **P < 0.01.

**Fig. S2.** Lung metastasis of AGS cells in *in vivo* metastatic model. (A) Representative images of lungs with metastatic nodules from mice in indicated groups (*n* = 3). (B) HE staining of lung tissues and the quantitative diagram of metastatic nodules (*n* = 3; one‐way ANOVA; **P < 0.01).
