# Supplementary figures and images for: FOXD1‐AS1 regulates FOXD1 translation and promotes gastric cancer progression and chemoresistance by activating the PI3K/AKT/mTOR pathway
Source: Mol Oncol. 2020 Nov 14;15(1):299–316. doi: 10.1002/1878-0261.12728 (PMC7782086; doi:10.1002/1878-0261.12728)

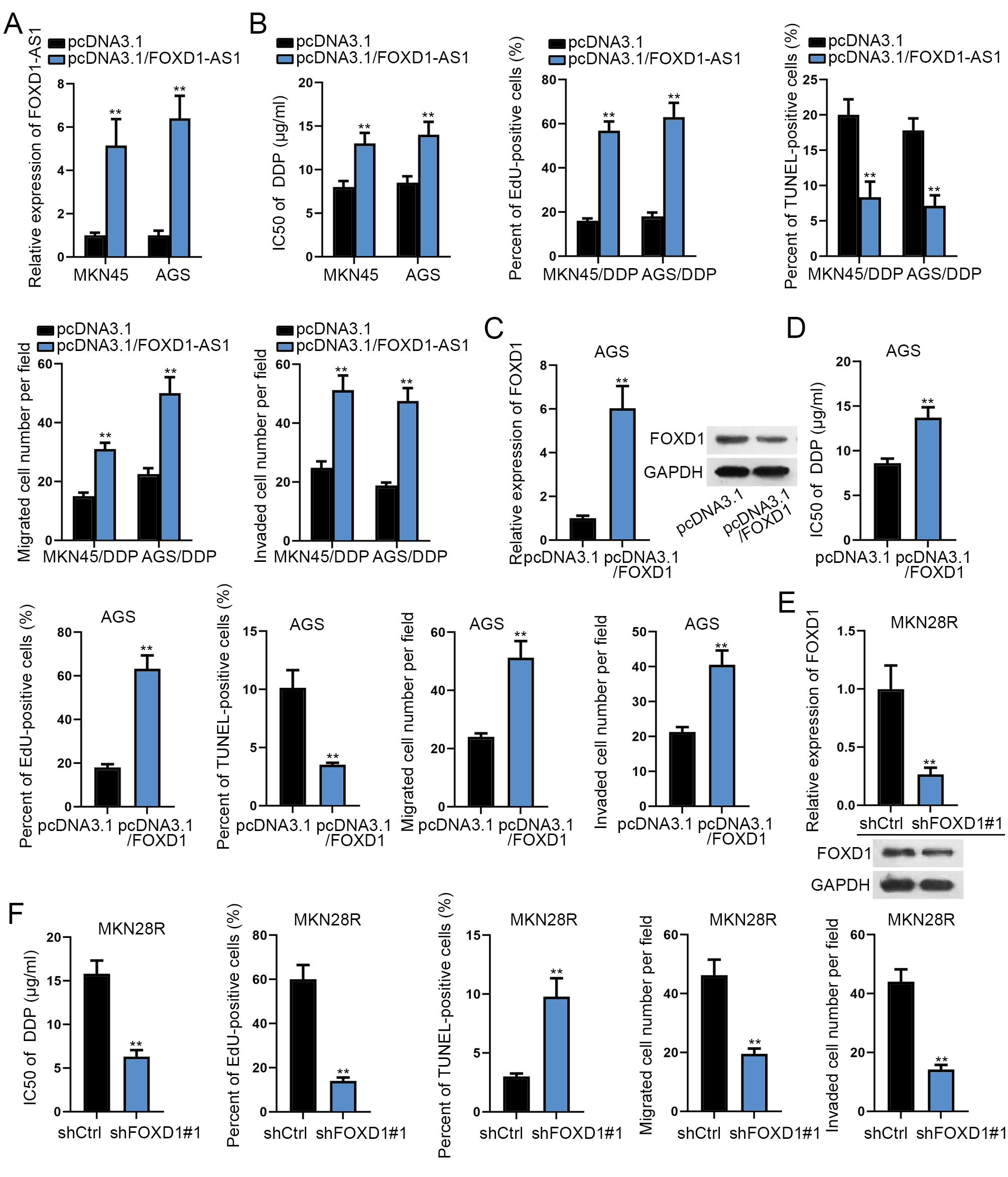

Supplement: Supplementary file 1 — Fig. S1. Impact of FOXD1‐AS1 or FOXD1 on the resistance of GC cells to DDP treatment. (A) FOXD1‐AS1 expression in MKN45 and AGS cells transfected with pcDNA3.1 or pcDNA3.1/FOXD1‐AS1 was assayed via qRT–PCR (n = 5, Student’s t‐test. (B) The effect of FOXD1‐AS1 upregulation on the resistance of MKN45 and AGS cells to DDP treatment was detected by CCK‐8, EdU, TUNEL, and Transwell assays (n = 5, Student’s t‐test). (C,D) The levels of FOXD1 in FOXD1‐overexpressed AGS cells were determined by qRT–PCR and western blot (n = 5, Student’s t‐test) (C). Its impact on DDP‐resistant AGS cells was tested by CCK‐8, EdU, and Transwell assays (D) (n = 5, Student’s t‐test). (E,F) FOXD1 expression in FOXD1‐depleted MKN28R cells were determined by qRT–PCR and western blot (E). Its impact on DDP‐sensitivity of MKN28R cells was estimated via CCK‐8, EdU, TUNEL, and Transwell assays (F) (n = 5, Student’s t‐test). Data are shown as mean ± SD (standard deviation). Error bars indicate SD. **P < 0.01. [file MOL2-15-299-s001.tif]

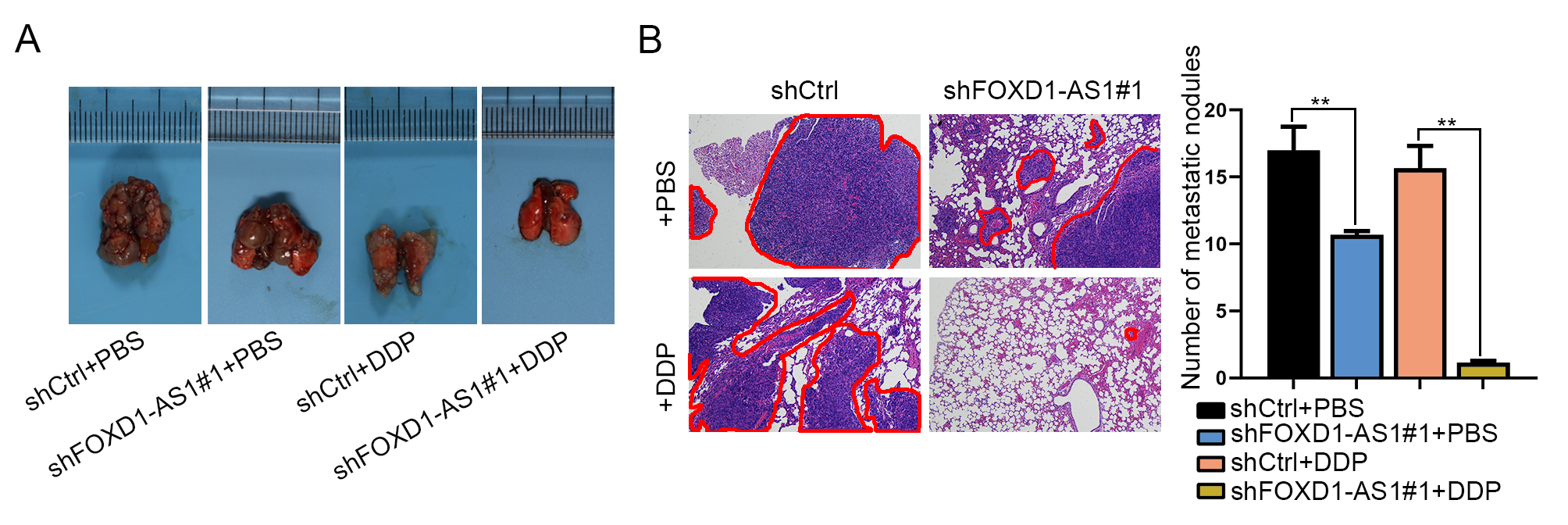

Supplement: Supplementary file 2 — Fig. S2. Lung metastasis of AGS cells in in vivo metastatic model. (A) Representative images of lungs with metastatic nodules from mice in indicated groups (n = 3). (B) HE staining of lung tissues and the quantitative diagram of metastatic nodules (n = 3; one‐way ANOVA; **P < 0.01). [file MOL2-15-299-s002.tif]
